# The birthing room and its influence on the promotion of a normal physiological childbirth - a qualitative interview study with midwives in Sweden

**DOI:** 10.1080/17482631.2021.1939937

**Published:** 2021-06-20

**Authors:** Anna Andrén, Cecily Begley, Helena Dahlberg, Marie Berg

**Affiliations:** a Institute of Health and Care Sciences, Sahlgrenska Academy, University of Gothenburg, Gothenburg, Sweden; b Department of Obstetrics, Sahlgrenska University Hospital, Region Västra Götaland, Gothenburg, Sweden; c School of Nursing and Midwifery, Trinity College Dublin, The University of Dublin, Dublin 2, Ireland

**Keywords:** Midwife, childbirth, birth environment, birthing room, hospital, salutogenesis, phenomenology, reflective lifeworld research

## Abstract

The birthing room is a major workplace for midwives but how it influences them in practice is not enough investigated.

**Purpose**: This study aimed to explore midwives´ experiences of how the birthing room affects them in their work to promote a normal physiological birth.

**Methods**: A phenomenological reflective lifeworld research approach was used and included individual interviews with 15 midwives working at four different hospitals in western Sweden, and of which two also assisted at homebirths. The analysis focused on the meanings of the study phenomenon.

**Results**: A birthing room can by its design either support a normal physiological birth or support a risk approach to childbirth. Four opposing constituents complete the essential meaning of the birthing rooms, and to which the midwives need to relate in their roles as guardians for normal birth: i) a private or a public room; ii) a home-like or hospital-like room; iii) a room promoting activity or passivity; iv) a room promoting the midwife´s presence or absence.

**Conclusions**: The birthing room mirrors a pathogenic-oriented care approach. A presupposition for the work to keep the birth bubble intact is to protect the mother from disturbing elements both inside and outside the room.

## Introduction

The environment in which health care occurs has substantial effects on patient’s health, safety and effectiveness of care (Ulrich et al., 2008). In maternity care, the birthplace has an impact on the birth outcomes and also on the extent to which medical interventions are being used. When comparing alternative versus conventional institutional settings for birth, the alternative settings are associated with increased spontaneous vaginal births, fewer instrumental births and episiotomies, less use of epidural anaesthesia, increased breastfeeding, and satisfaction with care (Hodnett et al., 2010). The same benefits have been reported in relation to the use of midwife-led continuity models of care (Sandall et al., 2016), which also suggests that midwives play an important role in promoting normal birth and ensuring the health of women and newborns in relation to childbirth.

For the majority of women in high- and middle-income countries, hospitals have become the “normal” place to give birth (Drglin, 2020). In Sweden, the place of this study, a majority of all women gives birth in hospitals since there are almost no other options available (Lindgren et al., 2014). This means that hospital birthing rooms have become the primary workplace for most midwives.

Research suggests that the design elements of hospital birthing rooms may obstruct and limit the midwife from conducting quality midwifery care. Bright lights, disturbing noise, and lack of privacy are some of the factors that for midwives may inhibit their work in promoting normal childbirth (Bourgeault et al., 2012; Davis & Homer, 2016; Hammond et al., 2014, 2017).

Even if the goal of Swedish maternity care is to promote normal physiological births, statistics show that births without medical interventions gradually decreased during recent decades. Between 1991 and 2017 the labour induction rates increased from 7.1 to 19.3%, the caesarean section rates from 10.9 to 17.3% (National Board of Health and Welfare, 2019), and in 2019, 57.6% of all first-time mothers received synthetic oxytocin for augmentation of labour (The Swedish Pregnancy Register, 2020). This development is similar to other high- and middle-income countries (World Health Organization, 2018) and raises questions on how to keep birth normal (International Confederation of Midwives, 2014).

Although some studies suggest that the birthing room impacts midwifery practice, there is still a lack of evidence on how the birthing room may influence the midwife to support normal birth, and in Sweden no such study has been conducted. Given the decline in normal births and that the hospital birthing room is the primary workplace for most midwives, it is important to investigate if there is a connection between these two variables.

## Aim

The aim of the study reported in this paper was to explore midwives´ experiences of how the birthing room affects them in their efforts to promote a normal physiological birth.

## Method

This study is part of the Room4Birth research project, which aims to extend knowledge on the design of the birthing room and its influence on labour and birth (Berg et al., 2019).

We used a qualitative phenomenological, reflective lifeworld research approach as it allows the researcher to explore a phenomenon in all its variations of meanings as well as its essential meaning structure (Dahlberg & Dahlberg, 2020). The phenomenon in this study is defined as: “midwives´ lived experiences of how the birthing room affects them in their efforts to promote and support a normal physiological birth.”

Sampling within a phenomenological approach is concerned with gathering richness and depth in experience and thus variations of experiences are searched. An important element in the search for meanings is the researcher’s presence and openness, which is obtained by having a bridling attitude all through the research process. When doing data collection through interviews, maintaining a bridling attitude implies that the researcher approaches the informants and their experiences in a questioning manner, in an unprejudiced way and with a self‐reflective attitude. During the analysis phase the bridling will keep the researcher present to the phenomenon in question and guide open reading and analysis; it obliges the researcher to problematize and reflect on taken-for-granted assumptions. This approach will allow the phenomenon in question to show itself in all its complexity and variations. Furthermore, the understanding and analysis process is slowed down, which facilitates the researcher to be open to the new and unexpected and let perhaps surprising meanings arise. The bridling process is not something that is performed once, but is an ongoing attitude throughout the whole process of planning the project, gathering and analysing data, and reporting the results (Dahlberg & Dahlberg, 2019, 2020; Dahlberg et al., 2008).

### Setting and participants

A strategic sampling strategy was used to ensure a varied selection of participants in order to get access to lived experiences of midwives of different ages, with different lengths of professional experience, and with different workplaces. Midwives from four different hospitals in western Sweden, who had worked in the labour ward for at least three years, were invited to participate in the study. Interested and selected midwives were contacted by email either by one of the researchers (AA, MB) or by the head of the unit in the department where they worked, with a request to participate in an individual interview. Those who showed interest in participating were contacted and given oral and written information about the study.

Eighteen midwives were asked to participate of which three declined due to lack of time. The fifteen participating midwives worked at four different hospitals and at six different labour wards. Two of the midwives also assisted at homebirths. Their age varied between 36–61 years old (median 48 years), and they had worked as midwives between 3–30 years, (median 14 years). Six of the participants worked in the ward where the Room4Birth study is taking place and had therefore experience of working in a newly built test room, designed to offer changes according to a woman´s wishes. These experiences of working in different birthplaces and settings, were considered beneficial for providing broad variations of lived experiences on how the birthing room affects the midwife in her work.

### Interviews

Each participating midwife received both verbal and written information about the study, and they were told that the interviews would be recorded, and that they could withdraw from the study at any time. Written informed consent was received prior to the interviews. Midwives were interviewed individually by either AA (n = 12), HD (n = 1) or MB (n = 2).

All participants decided time and place for the interview. Each interview began with an open-ended question: *how do you feel the birthing room affects your ability to promote a normal birth?* The participants were encouraged to elaborate on experiences and concrete situations related to the study phenomenon. Follow-up questions were asked continuously, such as: *can you give an example*? and *can you develop that more*? The interviews were recorded and lasted between 28–64 minutes.

### Analysis

The recorded interviews were transcribed verbatim and analysed according to the reflective lifeworld research approach. According to this approach, analysis is characterized by a constant movement between the whole-parts-the whole, focusing on meanings in each interview and the interview as a whole, as well as between each interview and all interviews together. The aim of the analysis was to explore and describe the essential structure of the phenomenon, which can be reached through understanding the variations of meaning. The process of analysis went from discovering meaning units to finding patterns of meaning, an essential structure, and finally, variations of the meaning patterns, described below as constituents (Dahlberg & Dahlberg, 2019, 2020; Dahlberg et al., 2008)

To begin with, each transcript was read repeatedly to get a sense of it as a whole. After this initial reading, a closer reading of each transcript was performed, and the data was divided into meaning units that answered the aim of the study. For the meaning patterns to emerge, we asked ourselves questions such as “What is being said about the phenomenon?” “What is the meaning of this?” and “Is this the actual meaning being expressed, or does it mean something else?”. What is it that we don’t see?”. Three of us researchers (AA, MB and CB) are midwives, which challenged us further to have an open attitude to the study phenomenon, instead of seeing what we usually see. As part of the bridling process, questions were repeatedly asked during the analysis about whether this was the best way to understand the phenomenon, or whether it should be understood differently. We also went back repeatedly to the original whole of interview texts, in an oscillation between the whole of the text, parts and a new whole that defined the essential meaning structure of the phenomenon.

Finally, the essential meaning that characterized the phenomenon was identified, and the clusters were abstracted to a higher level, grouped together, and made into constituents that described both the essential meaning and the phenomenon as a whole. This analysis was conducted by two of us (AA and MB), while HD and CB read, discussed and critiqued the emerging and final description, in the process known as “peer debriefing” (Lincoln & Guba, 1985).

### Ethical considerations

Ethical approval for the study was received from the Swedish Ethical Review Authority (Dnr 2020–01064). All procedures were in accordance with the 1964 Helsinki declaration and its later amendments (World Medical Association, 2013)

## Results

In the midwives´ descriptions, they appear as guardians of the normal, physiological and healthy birth. This is an intentional and essential act which the birthing room has the power, by its design and the activities in it, to either support or hinder by promoting a risk approach to childbirth. The labour wards in which the midwives worked appear to be organized mainly to reduce and illuminate risks and demonstrate the drive to have control over the birth process. This is obvious in written care plans in the medical records, and it is also mirrored in the design and physical interior of the birthing rooms.

Inside the birthing room, each mother’s well-being is central for the midwives. In their roles as guardians for normal, physiological, and healthy birth, they need to relate to, act, and co-operate with the room. This implies the need for them to adapt, change, reduce, and enhance the physical functions, and to eliminate disturbing activities that occur, both inside and outside the room. The midwives do not require anything for their own sake, all their efforts are directed towards making the birthing room into a supportive birthplace for the mother.

Four constituents, which consist of four pairs of opposing characteristics, complete this essential meaning of the birthing room for the midwives: i) a private or a public room; ii) a home-like or hospital-like room; iii) a room promoting activity or passivity; iv) a room promoting the midwife´s presence or absence.

In our descriptions the midwife is referred to as “she”, and the mother giving birth is also referred to as “she”.

### A private or a public room

One constituent structuring the meaning of the room concerns *privacy* and *publicity*. As guardians, the midwives want to encourage and promote the room to be private, thus protecting against publicity.

To make the birth public is the result of an institution focusing on risk, which includes the drive to have control over the process, thus allowing what is happening in the mother´s body to be available to everyone outside the room. This is the case with having Cardiotocography (CTG) monitors in the office, where everyone can observe what is going on inside the birthing room, with no need to step inside.

Besides this, the door has a central function on how the room is constituted in terms of keeping private or public. It is the way not only into and out of the birthing room, but gives access to the mother’s birth nest, to her space, her bubble, and to her body.

In a room supporting privacy the door is closed, which gives the childbirth process protection from the outside world and allows for the mother to be in her “birth body”—her “bubble”. A closed door creates an atmosphere of peace, security, and emotional safety. The mother is not disturbed by activities out in the corridor. The privacy is preserved by the midwives knocking and waiting for answers before entering. It is a way to demonstrate that the space inside belongs to the mother who then gets to welcome the staff in:

It is still their birth. We are the ones who are invited to the patient. We are involved as staff. And if you turn that perspective around a bit, it will be … the patient who should welcome us in for it to be really good. (P14)

I will step into the room with reverence, for the woman. It should be her domain. She should be like a queen to whom I, as a midwife, should treat myself reverently. She will decide how it will be around her. (P8)

In contrast, the door in a public birthing room is often open and the midwives, together with other staff at the ward, are often running in and out. It is like the room has no door, or that it is more like a revolving door, lacking handles. The staff have free access, they can just walk straight in. Also, the midwife and assistant nurse who work inside the room can easily move in and out, to pick up an aid, look after other mothers, join the rounds, or respond to an alarm. Every time the door opens, the world outside is remembered by those inside. It may be another light out there or other sounds that signal stress or danger. It can disturb the birthing process; the atmosphere and the bubble inside the birthing room blows up a little every time the midwife opens the door and goes out:

It is a very sensitive situation when a woman gives birth to a child, it has been known since time immemorial that when the birth was underway, the room was sealed, then anyone could not run in and out of there. It was they who were to take part in the birth and I have experienced that it still applies today, but we do not do that. (P4)

If she [the mother] can focus on giving birth to her baby, if she can be in that bubble that you need to be in when giving birth, then it is very conducive to that hormonal play and also her ability to be in the bubble with as few breaks as possible. I think you need to have this focus in your childbirth bubble. If I now constantly say “I’m just going to go out and get a thing”, you force her out of this bubble every time you do that. (P5)

To guard the normal, healthy, physiological birth in such a public room, including keeping the door closed, is hard work. Knowing that the door may swing open at any time, some midwives set up screens to further protect the mother´s privacy. They also try to warn the mother if someone is about to enter the room, i.e., a doctor or a colleague.

When something urgent happens, the door is wide open, everyone has access to it, and it is as though the door no longer exists. The room goes from being very private to becoming public. The mother then becomes exposed to all the people who run into the room. In these situations, one midwife describes that she may have to actively close the door to keep the staff out who are not needed at the moment. Otherwise, the situation may worsen:

And then I can only say: “No, stop”, at the door, “I do not want all of you in here. One person is enough now. We sound the alarm if we need more”. Because that’s also about integrity, calm. Because I think if you take in five, six people who start spinning around the room, what does it do to the woman and what does it do to her possible risk of bleeding? (P10)

The bathtub: Privacy and publicity are also influenced by whether there is a bathtub in the birthing room or if it is placed elsewhere. If there is no bathtub in the birthing room, a mother that wants to take a bath will have to change rooms. This may cause her to refrain from bathing since it means that she has to leave her private space, move out in the corridor and into a new environment and into a new room that does not belong to her:

Then you need to break this bubble in the room and go somewhere else, and if you go to another room I think many people also think “I have to go back from here later, back to the room where I will give birth” and then maybe you feel that you do not get any peace in it, “I have to leave here again, this is a bathroom ... I might take it up for someone else”. It’s easier to try it out too, it’s much easier if it’s there in your own room than to go through an entire corridor having pains, you may not be eager for that at all. (P5)

What is going on outside the window may also disturb the privacy of the room. Sound from an ambulance, people working just outside the window, or if there are other buildings nearby from which one can see straight into the room, may cause the mother to feel uncomfortable. Knowing that there are people outside that may see or hear you birthing can be inhibitory. For the room to stay private and for the bubble to stay intact, there can be no risk of being seen or heard from the outside:

Something I do not think contributes to that bubble is the environment outside the windows here. Partly that there are other windows enabling others to look into our windows even if far away … you may not feel completely protected … The integrity is disturbed by a crane outside the window that builds a new clinic next door. There is a truck driver two and a half meters outside … you might not want to share your birth with him. So, I think it bothers women, the environment outside the window. Or the feeling that you are not completely to yourself. (P3)

### A home-like or a hospital-like room

Next constituent is whether the room is *home-like* or *hospital-like*. A room that promotes childbirth is, according to the midwives, a room that provides emotional safety. And a room that feels emotionally safe is a room that feels homely. A hospital-like room is the opposite of home-like, it is a room that is designed according to the business and reflects the prevailing model of care and view of childbirth as a risk. Although it can never be like home in a hospital, there are aspects of the room that can either make it easier or harder for the midwives to create a homely environment for the mother.

Light, colours, nature, bedspread and personal items: One crucial element to make the room homely, is the lighting. If the lighting can be dimmed it creates a cozy, calm, and enclosing atmosphere. Earthy colours on the walls, nice pictures or green plants may also support a homely feeling. The environment should resemble a spa, *it gives a safe and nice feeling* (P12). In some hospitals, the birthing bed is covered with pretty duvet covers to make it more inviting. All such things help not only the mothers, but also the midwives to relax, which is positive for the birth process:

I think it [the room] feels cozier and safer if it is not a very strong lighting … I think that partly it is for her who gives birth, but it is also the case that it is calmer for me to work in. A very bright light signals that it is a bit urgent. (P5)

It is what the animals do, the mothers go to a safe place. If thunders and rains, or bombs or predators are coming to take them, then all contractions stop and they run and they wait until everything is calm and quiet and then let the babies out when they feel “Now I can let them out”. That’s how it is in the animal kingdom and I think it’s exactly the same, I do not let anything out until I feel that this is the right place. (P6)

Nesting and taking possession of the room: To create a homely feeling, some mothers bring own personal items from home such as photographs, pieces of fabric shawls, their own pillow, or speakers so that they can listen to their own chosen music. The midwives welcome and encourage this nesting behaviour. If the mother can feel like home in the birthing room, it facilitates for her to take possession of the room. She doesn’t need to behave, asking for the midwife’s permission, instead she moves around as she pleases and uses all corners of the space. The room becomes her domain, just like home and the relationship between the mother and the midwife become more equal. For the midwives who work with assisting home births, they are guests and the one needing to adapt to the mother´s and her family´s domain.

Then we have had couples here who have brought a lot of fabrics with them. They’ve hung things up. They have turned the lights off and were really nesting properly, so that they get what they want. And that is a great thing … Those couples have dared to take control, “We are the ones who come here. We are going to give birth. You will help us and support us”. They feel at home. (P13)

It should be equal, that they should be able to talk to me, how they want it, and dare to do it. And that it is their experience that I really only get to follow. And that’s how it is if they come and decorate the room the way they want. I step in with them, instead of them being with me. So then it will be a different atmosphere. It will in a way be more equal then. (P9)

[At a mother´s home] I adapt to the premises that are available. The person who is going to give birth does not have to adapt to me or the premises, because they are hers. She has an ownership there. (P8)

A hospital-like room, on the other hand, belongs to the staff, it is their domain. In such a room there will be more of a hierarchical order where the mother is subordinate to the midwife who controls and steers and focuses on performing her own duties. The midwives need to be in control and to be able to act quickly in case of emergency. There has to be enough floor space to move around, and a certain order—the cabinets should be marked with their contents, so it is easy to find items. If things are in the way of the midwife, she moves them:

Should there be an emergency situation, we must be free to move around in the room ... Because it will be in the way, I decide ... to take out the walking table. Now she does not have access to the walking table. And then I take in a Pilates ball and she has to sit on it for a while. So it will be me who decides. (P9)

Childbirth can be completely normal from the first moment until you leave the room, but urgent things can also suddenly happen. Completely unexpected … where you need to move beds quickly, have the opportunity to be around the woman from all directions, that you do not have the bed against a wall or that you do not have access to the things you need, if needed! (P6)

A hospital-like birthing room is designed from a risk perspective. Such a room does not support birth since it does not signal that birth can be something normal. Instead the medical equipment indicates that giving birth is a risky event. To have an emergency table for the newborn child inside the room may have some advantages but “*What does the table that stands there say? Well, something can happen to your child* (P10).”

The interior in such a room gives a sterile impression with fluorescent lamps in the ceiling giving a strong light that cannot be regulated. The walls are often white coloured, and there are very few items that soften the atmosphere and make the room inviting to stay in. The bed is made with just a sheet and a plastic protective mat placed on top, which leaves the mother to wonder if birth is a messy event.

It is precisely this medical, that you enter the birthing room and there is a lot of medical equipment in front, such as nitrous oxide tubes, CTG device, and drip stands. I think that is very negative. Because when you come in and are healthy and are about to give birth, it gives you the feeling that you are in hospital and … the feeling that a catastrophe can happen here. … this feeling is exactly the opposite of what you should feel when you go into the labour process, a sense of security. (P15)

Such a hospital-like room makes the mother feel like a guest and it can even trigger fear of giving birth. Because even if the midwife is trying not to *“intrude too much on the couple, as it is their room*. (P2),” it is difficult or even impossible for the mother to nest. Instead, they revert to the role of a patient—passive and docile—waiting for the midwife’s instructions and permission before doing anything such as changing position or going to the toilet:

I think that very many [women and their companions] find it very difficult to make it their own … because they are patients in hospitals. They come into a hospital room and we as staff have a position of power in there, we control the birth very much. (P1)

In the hospital-like room the midwives must work hard to make the mother feel emotionally secure. They try to normalize what looks deplorable by explaining all the medical equipment and buttons in the room—what it is and what it might be used for, so there are no strange things that can create fear. Some midwives move medical equipment out of the way when not needed and some encourage the mothers to spread their things out just like they do at home.

What I usually do, is that I explain when we enter the room what the different things are, so that they know. “That it’s not dangerous, it’s not strange, but this may be something you want eventually”. What the CTG device with the monitor is. That the computer is a work tool for me. That we have our baby’s table with a heat lamp over. And that we have an operating lamp in the ceiling if I need to suture after. Just so they know what it is they have around them. I think that creates more security than being thrown into a room where there are appliances you do not know what it is and what it is used for. (P11)

### A room promoting activity or passivity

This constituent concerns whether the birth room is promoting the mother to be more *active* or more *passive* during childbirth, both of which influence the midwives´ work. A room that promotes activity inspires the mother to movement. Preferably, it is the mother herself that takes the initiative to move and chooses which positions she wants to be in. This will strengthen her and give her influence over her birth, which may result in a better birth experience. In such rooms there is sufficient floorspace to move around. There are visible options, such as Pilates balls, walking trays, yoga mats, or a large bathtub inside the room. On the walls there may be pictures of different positions that can facilitate childbirth, which may serve as a source of inspiration. Since every mother is unique, the room offers several alternatives and adaptions:

That she has a freedom of choice to find something that suits her, so that she feels that it is her birth, that she owns it. That she can choose. Not just because I say “Now I think you should kneel in bed”, but “No, I might want to hang my birth support rope here by the door”. So that she can get ideas to have more influence over her own birth which can hopefully lead to her having a positive birth experience. (P13)

If you have things that promote that the woman is active, I think that it becomes a positive memory of being active in her birth, that it is not someone else who does it for you. My experience is that it can be experienced that someone else takes over, that you are just a machine that gets this child out! It’s like a human being giving birth to a child! For me it is an existential experience that I must be active to do myself, there is no one who can do it for me … And I think it promotes one’s experience, that “My body has given birth to children”. My goal as a midwife is for women to feel that “I did it!”, Not that I have done it for them. (P1)

The opposite, a room that is designed to promote passivity strengthens a risk perspective of childbirth. This room is not designed to encourage movement. In such a room the birth bed is the main feature, around which the midwives practice. Medical equipment, such as the CTG device, the nitrous oxide tubes, drip stands and infusion pumps, are placed around the head end of the bed. Its appearance, its placement, and the fact that there are few other comfortable options, such as a large bathtub or a sofa, indicates to the mother that the birth bed is where she should be. She becomes passive and in-active, and also more dependent on the midwives who in turn become more controlling in their efforts to encourage movement.

The descriptions show that the bed, for many midwives, is an important part of birthing. It is a central and safe place in the room and around which the midwives do their work such as comforting, connecting to CTG, performing vaginal examinations, observing the perineum during pushing, or managing a haemorrhage:

As a midwife, if you have the bed … It is very easy to say “Yes, but lie down on the bed, and we will start by doing the CTG”. That’s how you start, you almost put the patient into the bed. (P15)

… Then of course I want them in bed when they are about to give birth, so that I can see the perineum properly and see what the woman looks like and how the baby is coming. (P14)

However, the midwives’ experiences of the birth bed contain contradictions. At the same time that they express the need for the bed in certain situations, they struggle with it in others. Once the mother has ended up in the bed, it can be very difficult to get her out. Therefore, different techniques are used to prevent the mother from entering the birth bed: One midwife describes how she usually raises the bed to maximum before she lets the mother into the room. Others try to re-furnish the room and move the bed aside, hiding it, although it can be a risky event since interfering with the order in the room may cause tension between the midwife and other colleagues. Another midwife states that she avoids instructing a mother to lie down when performing certain interventions. For the midwives working with home births, a bed is not a central thing for their practice, it is mostly used after the child is born.

If I am to carry out various interventions, I try not to be affected by her position. I can check when the woman is standing up at the edge of the bed, if possible. It’s not always possible, but I can always try it first. It may not be that she has to go to bed. I can put on a scalp electrode when she stands up at the edge of the bed and you can listen to fetal sounds in all sorts of different positions. You can give an injection when she sits up and stands up and so on. Or lying in the bathtub. I never tell a woman to get out of the bath for me to examine, for example. (P1)

Generally, in a home birth, women do not use their beds until after the births … you are usually in the living room, the dining room, leaning over the table or leaning over the kitchen counter. Or lean back against the sofa, or you walk a lot more. And maybe most women who give birth at home nowadays want to bring a pool. You can see that when you have something as powerful as a birthing pool, it becomes the center of the room instead of the bed. But even at a home birth where there is no pool, I have never experienced that. The mother seeking out the bed. (P7)

### A room promoting the midwife´s presence or absence

This constituent describes how the birthing room, and its surroundings, may either promote or hinder the midwife to be present, including both physical and mental presence. It also describes how the midwife´s presence in the birth room affects the mother´s ability to be in her own body—the birth´s inner room.

Since the birth room at a hospital is not a separate unit, what is happening outside the room is somehow reflected on the inside. Much of the midwives´ *presence* and *absence* in the birthing room is influenced by activities out in the corridor, in the office or in another birthing room.

When the midwives´ presence is supported, the room provides space for them to just “be”. With their presence, the midwives signal that the mother is the focus of their attention. The midwives have little concern for their own comfort in the room, one midwife stated she didn´t need anything but “*maybe a small chair to sit on* (P13).” However, by sitting down calmly, the midwives with their body language can convey that everything is going well, that the process is normal, and nothing dangerous is about to happen:

If I can then sit on a Pilates ball and wiggle my toes and look generally calm, I can also convey that it is not dangerous because then I would not have been sitting here. Because then I would have been much more active in here. (P3)

When the midwives are present inside the birth room, they can gather information about the birth process, in order to discover small changes that can tell about what is happening in the woman´s body, they *“see and hear and listen and taste the woman all the time* (P11)”.

Presence enables the midwives to encourage and support the mother to focus on giving birth, to be present in her own birthing body, just go with the flow and find her inner security. To achieve this, the mother needs to understand what is happening, otherwise she may become frightened:

Because if she does not understand why she suddenly starts to feel a little poopy and thinks, “oh God, how embarrassing. This is very difficult. And I need to go poop”. And then comes this horror, “shall I poop on myself?” And so these negative fears are started in the body, which then suppresses the good hormones. Instead of understanding, “this is something natural. This is not something I need to be afraid of. It just tells me that the birth has gone very well, that this is happening, that I am starting to feel this way”. Then she has not become afraid of it and begun to tense up and work against her body. But it is also about seeing the woman’s body as a room. That this is where so much happens, in the woman’s body … not only the physical space around, which of course also affects. But so it’s about finding security in the body. (P11)

The room should also support the presence of the mother´s companion. He or she needs to have the space to support the mother, but also have room for rest and recovery. There should preferably be a place in the room where the couple can have a physical presence, for example, a sofa where they can sit together, since physical contact creates security and promotes oxytocin production.

Some [mothers] may have concern for their partner, that the partner should be comfortable. And if there is no place where he or she can lie down, they may find it unwelcoming and strange … for the partners are their security and it [the room] must be just as inviting for them. (P4)

The biggest hurdle to the midwives´ presence in the birthing room is that they have to take care of more than one mother in the labour and birthing process at the same time. The care is not organized in such a way that there is one midwife per mother giving birth. There are several physical attributes in the birthing room and on the ward that support and enable this care model to function.

One such physical attribute is the CTG device, with tracings that are displayed on a monitor, both inside the delivery room and outside in the office. The CTG monitoring can thus facilitate the midwives’ work because they can keep track of the baby’s health even if they are not in the room, but at the same time monitoring creates a culture that leads the midwives away from the birthing room. One midwife shared that she sometimes has more than one CTG on display inside a delivery room, which signals that she is divided:

I may have to have the other CTG curve [displayed] on her curve, so that I see both sometimes. And then they can also be stressed by the fact that they see that I have two that I must keep an eye on. That my attention is not only on them, but I must keep an eye on one more. (P13)

But it’s also this thing that here we are talking about how the health care system views a birth, right? The care clearly shows that the focus should be on the CTG graph because we can sit in the coffee room and look at our curves. It can take us away from the woman. We do not have to be in there to listen every 15 minutes. Because we can be in the coffee room and look at the screen instead. (P7)

Inside the birthing room, the CTG device takes focus away from the mother at the same time it prevents her from being present in her birth body. When everyone in the room looks at the CTG monitor, what happens there becomes the centre of the childbirth. Somehow what is shown on the monitor becomes more important than what the birth body is showing, which may cause the mothers to doubt their body and their ability to give birth. When that occurs, the midwives may need to actively turn the monitor away so that the mother does not see it. If the companions are too much focused on the CTG monitor, one midwife explained how she sometimes encourages them to put their hand on the mothers´ stomach instead and feel when the contraction comes:

And they say, “Here it [the contractions] comes. Here it comes. Here it comes.” And then I can sometimes show them how they can put a hand on their stomach and feel it coming. (P7)

Another physical attribute that discourages the midwives from being present in the room, both mentally and physically, is the alarm system. Inside each delivery room, there is an alarm system that those in the room can use to call attention and signal that they need help. In some cases, the alarms go out in viewfinders carried by each staff member, in other cases it rings on a telephone, or the room that alarms is shown on a display on the wall. The alarm system means that the staff hear all the alarms in the entire labour ward, even from the rooms for which they are not responsible, and the women they care for can also hear, which is disturbing for them:

There are different signals that penetrates the room. Maybe I have my viewfinder in my pocket that sounds out load and then they [the mother and her companion] ask me why it beeps, if the ward is very busy and if it is stressful outside the room. (P4)

The ringing of the alarm system is disturbing for the midwives as it indicates others’ needs in the wider labour ward. This can cause stress for the midwife, which can also be transmitted to the mother who may lose the concentration on her body and disturb her birthing bubble.

## Discussion

This study shows that the birthing room either supports or hinders midwives to do their job as guardians for a healthy, normal physiological birth. The birthing rooms have the power to optimize or hinder this work. The labour wards where the interviewed midwives worked were mainly organized to reduce risks and treat complications and were thus dominated by a pathogenic-orientation to care. This was also mirrored in the birthing rooms which had a clinical, disease-oriented design and interiors. There was often bright fluorescent light, the birthing bed was placed in the centre with medical equipment around it, and there were few elements that created a warm and inviting atmosphere.

A pathogenic oriented healthcare focusses on detecting and treating pathological conditions, and classifies patients as “healthy or not”, or as being at a certain risk level. This is the opposite to salutogenic oriented healthcare, which emphasizes that humans are continuously, in daily life, moving between the two endpoints dis-ease and ease/health, and that healthcare should support patients to grasp their general resistance resources that promotes a move towards health and well-being (Mittelmark et al., 2017).

Antonovsky, the father of the salutogenic theory, concluded already in the mid-1990s that society is entirely rooted in pathogenic thinking with disease prevention via risk factor reduction (Antonovsky, 1996). This echoes what Davis-Floyd also described about childbirth care at almost the same period, that a “technocratic paradigm of childbirth” is ruling in which there is a body/mind separation, the body is viewed as a machine, the patient is treated as an object, the organization is hierarchical, and the care is standardized, and also includes routine procedures that have no scientific evidence (Davis‐Floyd, 2001).

Antonovsky was convinced that the pathogenic orientation would continue to dominate the healthcare arena. To promote health optimally, his hope was that salutogenesis and pathogenesis would exist side by side on equal and complementary terms (Antonovsky, 1996). In our interviews with the midwives we did not, however, find that salutogenesis existed on equal terms with the pathogenic approach in the labour ward settings where they worked. Instead, we found that the midwives had a hard time navigating between their desired focus, salutogenic-oriented midwifery, and the dominating pathogenic-oriented care. By acting as guardians inside the birthing rooms, the midwives were the guarantee that the risk approach would not totally dominate to the detriment of the normal physiological process of childbirth.

Other studies have shown similar difficulties for midwives. An interview study with midwives working at a modern, centralized and high-technology hospital in Norway shows that there was an ideological battle in the birthing rooms between a biomedical and a sensual knowledge tradition, and which included a battle played out between active treatment and more natural treatment of childbirth. This battle affected the midwives’ way of doing midwifery, including their way of being in the birthing room and their thinking about the body and on safety (Blaaka & Schauer Eri, 2008). A medical approach to birth with a hierarchical system in which the birthing women were passive receivers of care, hindered midwives in an obstetric-led labour ward in Ireland from practicing true midwifery, and a normal birth option was rare (Keating & Fleming, 2009). Obstetric hospital settings in New Zealand have been found to make midwives fearful, which led them to adopt a medical care approach (Davis & Walker, 2010). In an ethnographic study using observation in birthing rooms at a hospital labour ward in Sweden, the midwives were seen to work in a “field of tension” in which they were caught between midwifery, medicine and the institutional bureaucracy, which in the end led them to doubt their own knowledge and also to lose their freedom to act as autonomous professionals (Nilsson et al., 2019).

The midwives in our study tried to manage these contradictory perspectives of care by adapting and changing the interior, and eliminating disturbing activities occurring both inside and outside the birthing room. These findings echo a study in Australia and the UK of midwives working in labour wards, birth centres and homes, and who considered it to be important to create the right ambiance and space conducive to birth. Even though an appropriate ambiance could be created in any birth setting, the hospital labour ward provided the most challenges, partly due to clinical features such as visible medical equipment and bright lights but also because of the noise and the lack of privacy for the birthing women (Davis & Homer, 2016).

One of the four opposing constituents completing the essential meaning of the birthing room for the midwives in our study was `a private or a public room´. Similar to the midwifery guardianship described in the theory of “Birth Territory” (Fahy & Parratt, 2006), the midwives described how they took on the role of guardians, protecting the mother from stressors both inside as well as outside the birthing room. As described in “Birth Territory”, the midwives tried to relieve the mothers from the responsibility of guarding their birthing place themselves, allowing them to stay focused in their bubble and in their body. For the midwives, protecting the mothers´ privacy meant always keeping a watchful eye on the door, controlling their own, as well as others,’ movements in and out the room, trying to keep the bubble intact. However, it was a challenge to keep the room private. There were no clear boundaries between private and public spaces. Even if there was a door, it functioned more as a revolving door, and everybody had access to the room and thus to the mother´s body. This fact concurs with an Australian study, which concludes that the organization holds the ultimate control over the birth space and they are only “lending” it to the women (Seibold et al., 2010). Midwives, however can, and do control privacy by ensuring the door is kept closed (Bourgeault et al., 2012).

The next opposing constituent about the meaning of the birthing room was whether it was `home-like or hospital-like´. To feel “at home” in the birthing room for a mother in birth implies that she can relate to it as her own, having the freedom to use it as she wishes without concern for other´s opinions. This can, according to Roxberg et al, be difficult to achieve for many women since the private space existing in one´s home is lost when entering the hospital environment (Roxberg et al., 2020).

For the birthing room to become more home-like, the midwives encouraged a “nesting” behaviour, which meant that the mother and her companion arranged the birth room to their preferences aiming at creating an emotionally safe birthplace. A mother´s nesting-behaviour during birth has been found (Walsh, 2006) to include her need to have a sense of emotional safety. Looking back at evolutionary history, choosing a familiar and non-threatening place for birth has been observed among female primates, a behaviour that can be applied to humans as well (Trevathan & McKenna, 1994). As our study highlights, promoting normal birth in a room that is hospital-like, where the mother is in a non-familiar environment, surrounded by non-familiar people, is a challenge of the utmost severity since this way of birthing is highly unnatural. Midwives in a Canadian study, similar to the midwives in our study, have described how the birthing space can be adapted by for example, modifying the lighting and using the bed for everyone to sit on (Bourgeault et al., 2012). A critical interpretive synthesis of 27 qualitative studies has concluded that one needs to create a homely, spiritual, safe and territorial space around childbirth (Carlsson et al., 2020), all facets of the two constituents above.

The third opposing constituent forming part of the essential meaning of the birthing room for the midwives in our study was whether the `room promoted activity or passivity´. In general, the midwives felt that the unfamiliar, sterile, and high-tech environment in the birthing room could cause mothers to become docile and passive. The birthing bed took over as the main focus, encouraging women to lie down in labour. For some midwives, the birth bed had become the safe place in the room, around which they conducted care. The bed was used for convenience. This is contrary to the global recommendation for the use of upright positions to enhance normal labour (Zang et al., 2021). Several midwives in our study found the birthing bed to be a major obstacle in their work to promote normal birth and they were aware that they sometimes steered women to the bed. This was especially stressed by the two midwives who also assisted at home births, when they rarely used the bed before the child was born. Contradictions regarding the use of the birth bed have also been described by midwives working in a public hospital in Australia. Midwives who had experience from an out of hospital setting, considered the bed as something to be avoided while midwives that did not have that experience regarded the birthing bed as an object necessary to undertake their job safely (Townsend et al., 2016). Having the experience of how women having home births interact with their natural environment may provide midwives with a different perspective (Lepori, 1994).

The fourth and final opposing constituent about the meaning of the birthing room was whether it was a room promoting the midwife´s “presence or absence´. The midwives” own comfort in the birthing room has, in an Australian study, been highlighted as a factor that may influence her presence in the birthing room. Lack of privacy and personal space can make the midwife more willing to leave the woman and sit in another part of the ward (Hammond et al., 2014). The midwives in our study expressed no concern for their own comfort in the birthing room but having a place to sit down and looking relaxed was an important part of reassuring the mother that they had time and were present, also a way to show that the birth process was normal. The midwives were also sometimes prevented from being present, due to having to care for more than one mother in labour. It is logical that heavy and fragmented workload can make it difficult for midwives to provide continuous presence in the birthing room, which has also been found in a Norwegian study (Aune et al., 2014).

A main finding in our study was that the medical equipment such as the CTG and the alarm system interfered with the midwives´ presence in the birthing room. The CTG compensated for the midwives´ presence in terms of observation of the foetal condition, but also made the mother public by putting the workings of her body on display in the office, allowing all staff to keep track of some signs of the birthing process. As described in an ethnographic study from the UK (Lankshear et al., 2005), many of the decisions in a labour ward setting revolve around interpretations of the CTG and are made by staff outside the birthing room. Mothers´ lack of influence and participation in decisions became evident in our study in the midwives´ descriptions about how they were torn between the birthing room and the office, and often had to leave the mother to join “the rounds” in the office outside. These findings are similar to what were found in an ethnographic study in Sweden; that many of the decisions important for the birthing women are made in the office, which also serve as a breathing space for midwives who, while chatting with their colleagues, can observe the CTG on the monitors (Nilsson et al., 2019).

### Strengths and limitations

For us three midwives we tried hard to reach a high level of self-awareness about our preunderstanding, and according to the method—to avoid a risk of bias—adopted a “bridling” attitude throughout the whole process. This approach increases the possibility that the researcher remains open to the studied phenomenon (Dahlberg et al., 2008). A strength of our study is that the analysis has been a multidisciplinary collaboration and, importantly, that one team member has a philosophical perspective and is not a midwife. This further deepens the understandings of the study phenomenon.

The midwives gave rich descriptions of their experiences; however, a limitation of the study is the generalizability, as it comprises the experiences of a small group of midwives working at only six labour wards in Sweden. In addition, six of the 15 participants had experience of the Room4Birth adaptable birthing room, so the extent, scope and range of their descriptions were probably greater than any other group of midwives would have had. This improves the scale, breadth and detail of the study. We do not suggest that the participants are representative of all midwives and all labour wards in Sweden, or in other countries. However, we believe that findings can be applicable in similar contexts

## Conclusion

Our interview study shows that the birthing rooms, as part of labour wards, have the power to optimize or hinder midwives to do their job as guardians for a healthy, normal physiological birth. Most birthing rooms mirror a pathogenic-oriented care approach, and such rooms make it more difficult for midwives to protect the women so they can remain in an intact birth bubble. To counteract these effects, midwives need to be present, to make the room private and home-like, to support the mothers to be active, and to protect them from disturbing elements both inside and outside the room.

## Clinical implications

Our study points to several areas that may be improved to facilitate midwives to promote a normal physiological birth in birthing rooms in hospital labour wards:

- Labour ward stake holders need to think more deeply about the possible effects of the birthing room environment on women and consider this also when renovating or building new birth settings.
- The disadvantages of clinical and medical equipment, including the birthing bed, being prominently displayed in each birthing room should be noted, and a means of concealing them safely should be considered.
- Efforts to ensure privacy and calm in birthing rooms should be encouraged by senior labour ward midwives.
- Improvements to the alarm system in labor wards need to be urgently discussed, to avoid the situation where midwives caring for one woman are drawn into emergency situations elsewhere in the ward, thus disturbing the calm and peace of the laboring woman in their care.
- To strengthen midwifery practice in risk-focused maternity care, there need to be guidelines that also support salutogenically-focused midwifery care.
